# MPGES-1-derived PGE2 suppresses CD80 expression on tumor-associated phagocytes to inhibit anti-tumor immune responses in breast cancer

**DOI:** 10.18632/oncotarget.3581

**Published:** 2015-03-14

**Authors:** Catherine Olesch, Weixiao Sha, Carlo Angioni, Lisa Katharina Sha, Elias Açaf, Paola Patrignani, Per-Johan Jakobsson, Heinfried H. Radeke, Sabine Grösch, Gerd Geisslinger, Andreas von Knethen, Andreas Weigert, Bernhard Brüne

**Affiliations:** ^1^ Institute of Biochemistry I, Faculty of Medicine, Goethe-University Frankfurt, Frankfurt, Germany; ^2^ Institute of Clinical Pharmacology/ZAFES, Faculty of Medicine, Goethe-University Frankfurt, Frankfurt, Germany; ^3^ Department of Neuroscience, Imaging and Clinical Sciences and Center of Excellence on Aging (CeSI), “G. d'Annunzio” University, Chieti, Italy; ^4^ Department of Medicine, Rheumatology Research Unit, Karolinska Institutet, Stockholm, Sweden; ^5^ Pharmazentrum Frankfurt/ZAFES, Faculty of Medicine, Goethe-University Frankfurt, Frankfurt, Germany

**Keywords:** prostaglandins, microenvironment, macrophage polarization, costimulation, cytotoxicity

## Abstract

Prostaglandin E_2_ (PGE_2_) favors multiple aspects of tumor development and immune evasion. Therefore, microsomal prostaglandin E synthase (mPGES-1/-2), is a potential target for cancer therapy. We explored whether inhibiting mPGES-1 in human and mouse models of breast cancer affects tumor-associated immunity. A new model of breast tumor spheroid killing by human PBMCs was developed. In this model, tumor killing required CD80 expression by tumor-associated phagocytes to trigger cytotoxic T cell activation. Pharmacological mPGES-1 inhibition increased CD80 expression, whereas addition of PGE_2_, a prostaglandin E2 receptor 2 (EP2) agonist, or activation of signaling downstream of EP2 reduced CD80 expression. Genetic ablation of mPGES-1 resulted in markedly reduced tumor growth in PyMT mice. Macrophages of mPGES-1^−/−^ PyMT mice indeed expressed elevated levels of CD80 compared to their wildtype counterparts. CD80 expression in tumor-spheroid infiltrating mPGES-1^−/−^ macrophages translated into antigen-specific cytotoxic T cell activation. In conclusion, mPGES-1 inhibition elevates CD80 expression by tumor-associated phagocytes to restrict tumor growth. We propose that mPGES-1 inhibition in combination with immune cell activation might be part of a therapeutic strategy to overcome the immunosuppressive tumor microenvironment.

## INTRODUCTION

Tumor-associated inflammation is known to drive carcinogenesis and thus, appears as a rational pharmacological target. In line, new chemotherapy concepts aim at *de novo* triggering and/or restoring immunological responses against tumors [1, 2]. In addition, activation of tumor-infiltrating phagocytes by toll-like receptor (TLR) agonists emerged as a promising therapeutic option [2, 3]. Phagocyte activation not only promotes tumor-associated antigen presentation and upregulates costimulatory B7 family molecules such as CD80/CD86 [4, 5] to trigger anti-tumor lymphocyte activation, but also initiates counter-regulatory signals that attenuate activation. Counter-regulatory mediators that are part of the activation-induced inflammatory process unfortunately drive carcinogenesis through various pathways [4, 6, 7]. This process is often overlooked during immunotherapy, since therapy-inflicted inflammation is primarily considered as a sign of successful immune activation. One mediator of tumor-promoting inflammation is prostaglandin E2 (PGE_2_). PGE_2_ is produced by cyclooxygenases (COX-1/-2) and downstream cytosolic or microsomal prostaglandin synthases (cPGES, mPGES-1/-2) [8]. Under inflammatory conditions, the enzymes cyclooxygenase-2 (COX-2) and microsomal prostaglandin E synthase-1 (mPGES-1) are rapidly induced and functionally couple to accomplish PGE_2_ synthesis [9, 10]. Inhibition of COX-2 with the intention to block PGE_2_ formation is widely used to limit inflammation and pain and has lately been proposed for treating or preventing cancer. However, COX-2 inhibition affects other prostanoids besides PGE_2_. This prompted the development of mPGES-1 inhibitors to selectively target PGE_2_ [11]. The role of COX-2-derived PGE_2_ in the pathogenesis of cancer is well characterized. It acts as a tumor cell survival/proliferation factor, promotes angiogenesis, and modulates immune responses [12, 13]. Regarding immune regulation, PGE_2_ favors the generation of human and murine myeloid-derived suppressor cells (MDSC), inhibits cytotoxic T cells (CTLs), and suppresses phagocyte activation and/or maturation under inflammatory conditions [8, 14-16]. However, only limited information is available whether and how PGE_2_ regulates the crosstalk of phagocytes and T cells in the tumor microenvironment. In addition, it is unclear whether mPGES-1 inhibition fully recapitulates effects of COX-2 inhibition, which so far is widely used to investigate the impact of PGE_2_ on immune cell function.

To address these questions, we used a direct human PBMC - 3D tumor spheroid co-culture system to mimic human breast tumor development [17]. First, we explored whether this experimental set up can be used to monitor immune cell - tumor interactions and defined conditions that are required to mount an anti-tumoral response. As markers of such an immune response we analyzed shrinkage of tumor spheroids, CD80/CD86 expression on antigen-presenting cells (APCs), and granzyme B (GrB) expression by CTLs [18, 19]. These markers were altered upon modulating PGE_2_ production and signalling. Second, we investigated the impact of mPGES-1 during breast cancer development in mice expressing the polyoma middle T oncogene (PyMT) under the control of the mouse mammary tumor virus (MMTV) promoter, which induces spontaneous mammary tumors [20]. Reduced growth of mPGES-1^−/−^ tumors was correlated to altered phagocyte activation, which we linked to CTL activation *in vitro*. Finally, we discuss potential therapeutic implications that arise from our data.

## RESULTS

### CD80 expression on phagocytes induces GrBhi CTLs and tumor killing

**Figure 1:**
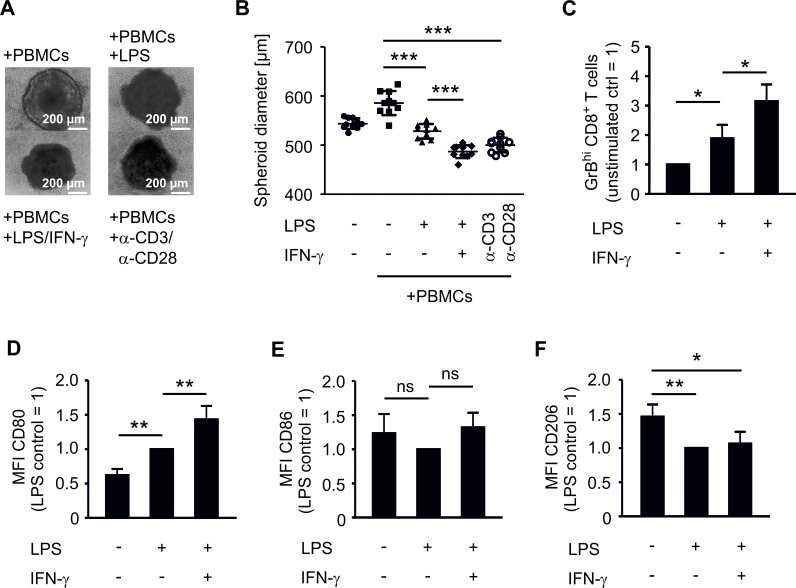
PBMCs require activation to restrict tumor spheroid growth and to upregulate activation markers PBMCs were pre-stimulated for 30 min as indicated and then cocultured with MCF-7 tumor spheroids for two days. Spheroid sizes were assessed by microscopy. (A) Photographs of PBMC-spheroid cocultures and (B) diameters of at least nine tumor spheroids are displayed. Data are representatives of three independent experiments. (C) Normalized ratio of GrB^hi^ CTLs after two days of coculture is displayed. Data are means ± SEM of at least four independent donors. Normalized (D) CD80, (E) CD86, and (F) CD206 expression on CD14^+^ CD11c^+^ phagocytes is displayed. Data are means ± SEM of at least four independent donors. *p*-values were calculated using one-way ANOVA (B-F) with Bonferroni's correction. Asterisks indicate significant differences between experimental groups (*, *p* ≤ .05, **, *p* ≤ .01, ***, *p* ≤ .001).

**Figure 2:**
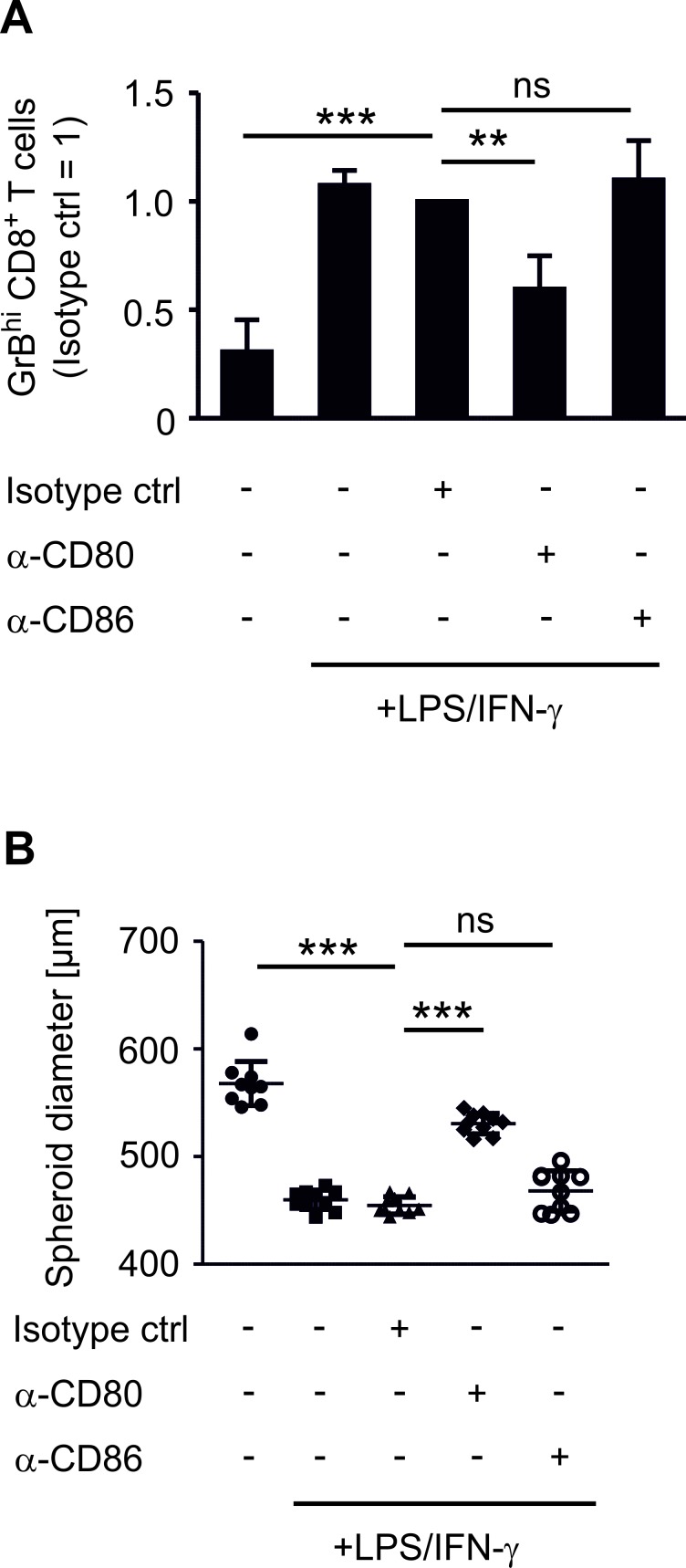
CD80 is needed for the induction of GrB^hi^ CTLs and tumor spheroid killing PBMCs were pre-stimulated for 30 min as indicated and subsequently cocultured with MCF-7 tumor spheroids for two days. (A) Normalized ratio of GrB^hi^ CTLs after two days of coculture is displayed. Data are means ± SEM of at least four independent donors. (B) Diameters of at least nine tumor spheroids are displayed. Data are representatives of three independent experiments. *p*-values were calculated using one-way ANOVA with Bonferroni's correction. Asterisks indicate significant differences between experimental groups (**, *p* ≤ .01, ***, *p* ≤ .001).

We compared the impact of COX-2 versus mPGES-1 inhibition on human experimental tumor-associated immunity in an authentic *in vitro* tumor model composed of human MCF-7 breast cancer cells and human PBMCs. Culturing human PBMCs from healthy donors with MCF-7 breast cancer tumor spheroids was expected to result in spontaneous allogeneic responses [26]. Surprisingly, this was not the case. PBMCs required addition of lipopolysaccharide (LPS) and interferon-γ (IFN-γ) or anti-CD3/anti-CD28 beads, i.e. immune cell activation, to reduce tumor spheroid size (Figure 1A,B). Challenging tumor spheroids with LPS in the absence of PBMCs did not affect spheroid sizes (data not shown). Addition of IFN-γ augmented the LPS-induced anti-tumoral activity of PBMCs, followed by spheroid size reduction (Figure 1A,B). Stimulation with anti-CD3/anti-CD28 beads, resulting among others in high IFN-γ production, was equally effective (Figure 1A,B). These data suggest that human PBMCs require a strong activating stimulus to overcome the suppressive tumor microenvironment. The tumor microenvironment was suppressive even in our MHC mismatched setting and in the presence of danger-associated molecular patterns derived from necrotic cells in the spheroid core [21]. Next, we explored markers of immune cell activation that could be used to monitor an efficient immune response against tumor spheroids. LPS increased the number of GrB-expressing (GrB^hi^) CTLs (Figure 1C, Figure S1A). This was further increased by IFN-γ (Figure 1C). Thus, a cytotoxic T cell response correlated with PBMC-mediated reduction of tumor spheroid size. Next, we investigated the phagocyte phenotype that was required for CTL activation. LPS-activated CD14^+^ CD11c^+^ human phagocytes expressed significantly higher levels of the inflammatory macrophage/DC marker CD80 in tumor spheroids, which again further increased upon addition of IFN-γ (Figure 1D, Figure S1B). In contrast, CD86 expression was not significantly altered after LPS or LPS/IFN-γ challenge (Figure 1E). Expression of CD206, a marker of anti-inflammatory macrophages, was significantly reduced upon LPS stimulation (Figure 1F). Next, we asked whether CD80 expression on phagocytes was a prerequisite for CTL activation and tumor spheroid size reduction. Therefore, CD80 on spheroid-infiltrating phagocytes was blocked using an anti-CD80 antibody. This treatment indeed reduced the numbers of GrB^hi^ CTLs and blunted tumor spheroid killing (Figure 2A,B). Neither a CD86-interfering antibody nor the isotype control affected numbers of GrB^hi^ CTLs or tumor spheroid killing (Figure 2A,B).

### Activation of PBMCs induces COX-2/mPGES-1-derived PGE2 in MCF-7 spheroid cocultures

**Figure 3:**
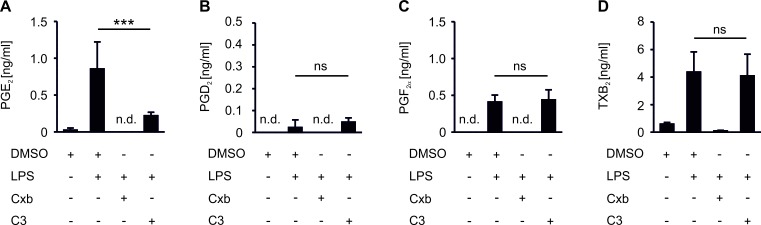
COX-2/mPGES-1-derived PGE_2_ production in PBMC spheroid cocultures PBMCs were pre-stimulated for 30 min as indicated and subsequently cocultured with MCF-7 tumor spheroids for two days. Cocultures were challenged with the COX-2 inhibitor celecoxib (CxB) or the mPGES-1 inhibitor C3. Prostanoids (A) PGE_2_, (B) PGD_2_, (C) PGF_2_α, and (D) TxB_2_ in supernatants of PBMC MCF-7 spheroid cocultures were measured by LC-MS/MS. Data are means ± SEM of at least four independent donors. *p*-values were calculated using one-way ANOVA with Bonferroni's correction. Asterisks indicate significant differences between experimental groups (***, *p* ≤ .001).

We asked whether PGE_2_ affects immune cell activation in our *in vitro* tumor killing model. Importantly, although 3D assembly of cancer cells can trigger expression of the PGE_2_ producing machinery, e.g. COX-2 [21], MCF-7 spheroids neither upregulated COX-2 mRNA nor produced PGE_2_ (Figure S2A,B). When PBMCs were cocultured with MCF-7 breast cancer spheroids, production of relevant PGE_2_ amounts was absent (Figure 3A). However, activation of PBMCs with LPS triggered the synthesis of PGE_2_. We also noticed the formation of other prostanoids such as PGF_2_α and thromboxane (TxB_2_), whereas the production of PGD_2_ remained low (Figure 3A-D). Apparently, a significant inflammatory milieu is required to trigger PGE_2_ accumulation. We then asked whether selective reduction of PGE_2_ (mPGES-1 inhibition) as opposed to blocking total prostaglandin production (COX-2 inhibition) modulates markers of tumor killing in the 3D model. Inhibition of both COX-2 with celecoxib (Cxb) and mPGES-1 with C3 indeed impaired PGE_2_ production in PBMC - tumor spheroid cocultures (Figure 3A). Importantly, whereas Cxb inhibited the production of all prostanoids tested, C3 selectively affected PGE_2_ (Figure 3A-D). Shunting of arachidonic acid towards the production of other prostaglandins did not occur [11].

### PGE_2_ limits CD80 expression by tumor-associated phagocytes via EP2 and cAMP

**Figure 4:**
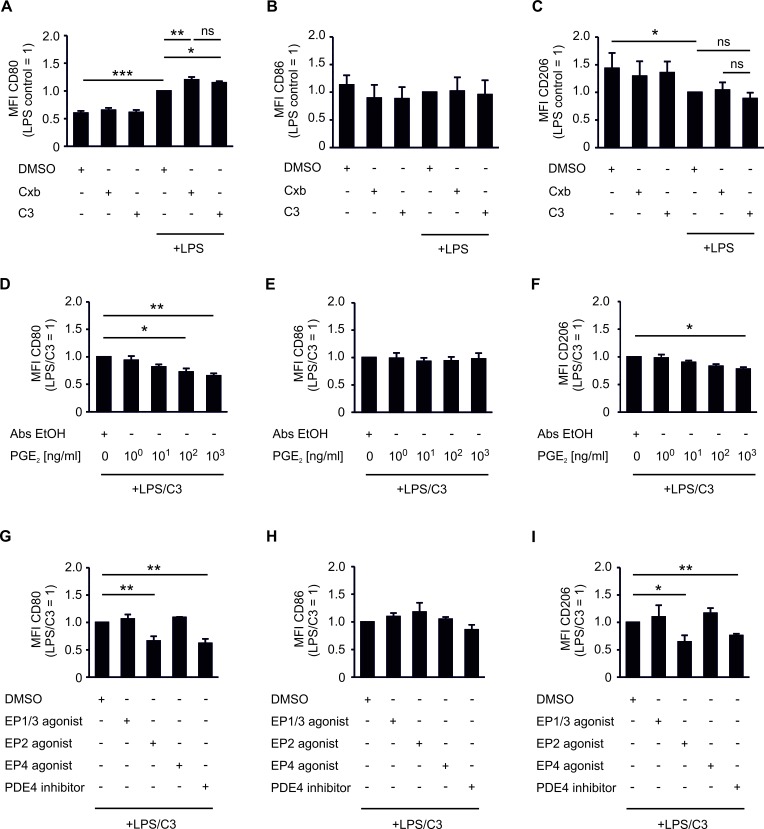
PGE_2_ inhibits CD80 and CD206 expression of CD14^+^ CD11c^+^ phagocytes PBMCs were pre-stimulated for 30 min as indicated and subsequently cocultured with MCF-7 tumor spheroids for two days. (A-C) Cocultures were challenged with the COX-2 inhibitor celecoxib (CxB) or the mPGES-1 inhibitor C3. (A) CD80, (B) CD86, and (C) CD206 expression of CD14^+^ CD11c^+^ phagocytes is displayed. Data are means ± SD of at least four independent donors. (D-F) Cocultures were dose-dependently challenged with authentic PGE_2_. (D) CD80, (E) CD86, and (F) CD206 expression of CD14^+^ CD11c^+^ phagocytes is displayed. Data are means ± SEM of at least four independent donors. (G-I) Cocultures were challenged with the EP1/3 agonist sulprostone, the EP2 agonist butaprost, the EP4 agonist cay10580, or the PDE4 inhibitor rolipram. (G) CD80, (H) CD86 and (I) CD206 expression of CD14^+^ CD11c^+^ phagocytes is displayed. Data are means ± SEM of at least four independent donors. *p*-values were calculated using one-way ANOVA with Bonferroni's correction. Asterisks indicate significant differences between experimental groups (*, *p* ≤ .05, **, *p* ≤ .01, ***, *p* ≤ .001).

Next, we compared the potency of Cxb and C3 to affect phagocyte CD80 expression, which was linked to spheroid killing (Figure 2). Cxb further increased CD80 expression in LPS-challenged tumor-associated phagocytes (Figure 4A). C3 raised LPS-induced CD80 expression to a level comparable to Cxb (Figure 4A). This suggested PGE_2_ as a prostanoid responsible for modulating CD80 expression on tumor-associated phagocytes. Incubation of non-activated PBMC spheroid cocultures with Cxb and C3 failed to enhance CD80 expression (Figure 4A). CD86 and CD206 expression were not significantly altered by Cxb or C3 (Figure 4B,C). Apparently PGE_2_, but not other prostanoids, selectively blocks activation-induced expression of the co-stimulatory molecule CD80. Importantly, authentic PGE_2_ dose-dependently counteracted LPS/C3-induced CD80 expression, which was significant at concentrations of 100 ng/ml (Figure 4D). CD86 expression was not affected (Figure 4E). Surprisingly CD206 expression was also inhibited by PGE_2_ (Figure 4F). Thus, PGE_2_ may impair polarization of tumor-associated phagocytes in general. PGE_2_ signals through four specific receptors (EP1-4), for which selective agonists are available. Application of the EP2 agonist butaprost, but neither the EP4 agonist cay10580, nor the EP1/3 agonist sulprostone, blocked LPS/C3-induced CD80 expression (Figure 4G). EP2 activation did not affect CD86 expression on phagocytes. However it significantly reduced CD206 expression (Figure 4H,I). EP2 triggers intracellular cAMP accumulation, which is simultaneously degraded by phosphodiesterase-4 (PDE4). Accordingly, increasing intracellular cAMP with the PDE4 inhibitor rolipram significantly counteracted LPS/C3-triggered CD80 expression (Figure 4G), without affecting CD86 (Figure 4H), but reducing CD206 expression (Figure 4I). Taken together, signaling via the EP2/cAMP-pathway counteracted LPS/C3-induced CD80 expression in tumor-associated human phagocytes and reduced the expression of the anti-inflammatory macrophage marker CD206.

### mPGES-1-deficiency delays tumor development *in vivo*

**Figure 5:**
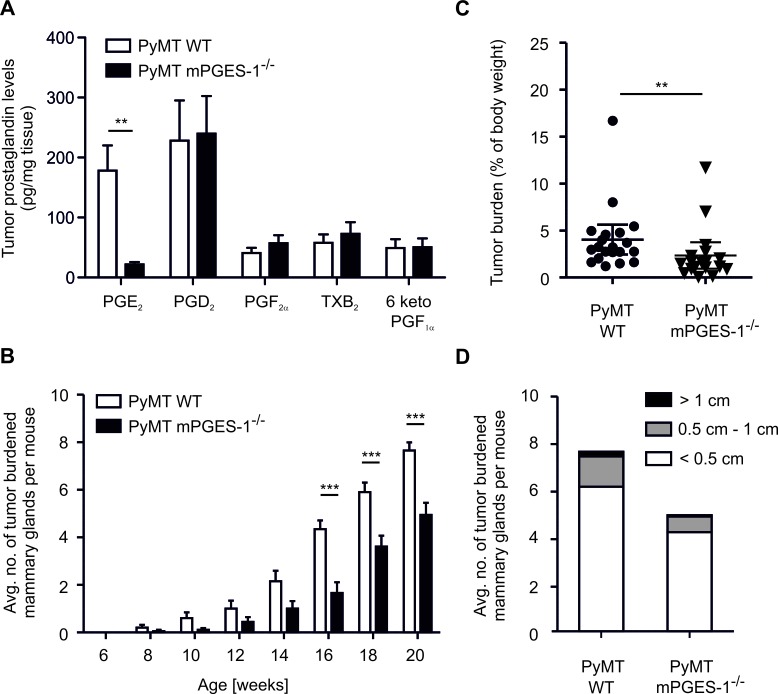
mPGES-1-deficiency impairs PyMT tumor growth (A) Prostanoids PGE_2_, PGD_2_, PGF_2_α, TxB_2_, and 6 keto PGF_1_α in wildtype (WT; n=8) and mPGES-1^−/−^ (n = 10) PyMT tumors were measured by LC-MS/MS. Data are means ± SEM. (B) The average number of tumor-bearing mammary glands per mouse is displayed. (C,D) PyMT mice were sacrificed 20 weeks after birth. (C) Tumor burden is defined as the ratio of total tumor mass and mouse body weight. (D) Tumors were categorized and their respective numbers are displayed. (B-D) Data are means ± SEM of 20 WT and 18 mPGES-1^−/−^ mice. *p*-values were calculated using student's t-test (C) or two-way ANOVA with Bonferroni's correction (A,B). Asterisks indicate significant differences between experimental groups (*, *p* ≤ .05, **, *p* ≤ .01, ***, *p* ≤ .001).

We asked whether regulation of parameters affecting tumor killing in the human *in vitro* setting could be recapitulated in a breast cancer model *in vivo*. We crossed mPGES-1-deficient mice into the PyMT background, to generate female mice that develop spontaneous breast cancer [20]. Tumors were first observed 8 weeks after birth and tumor development was monitored until sacrifice. MPGES-1-deficiency resulted in strongly reduced PGE_2_ levels in tumors after sacrifice (20 weeks) (Figure 5A). Importantly, shunting of arachidonic acid towards the production of other prostaglandins was also excluded in PyMT tumors (Figure 5A). Accordingly, both COX-1 and COX-2 mRNA were expressed in PyMT tumors, independent of the mPGES-1 status (Figure S3A). Lack of mPGES-1 delayed tumor development and reduced numbers of tumor-bearing mammary glands per mouse compared to WT PyMT mice (Figure 5B). After 20 weeks, loss of mPGES-1 still resulted in significantly reduced tumor mass (Figure 5C). However, the relative distribution of tumor size in mPGES-1-deficient PyMT mice was not different from WT PyMT mice (Figure 5D). Apparently, the absence of PGE_2_ enhances tumor dormancy rather than affecting tumor growth kinetics. This observation might indicate a role of mPGES-1-derived PGE_2_ in modulating tumor immune escape [27].

### Tumor-associated macrophage content and activation is altered in mPGES-1^−/−^ tumors

**Figure 6:**
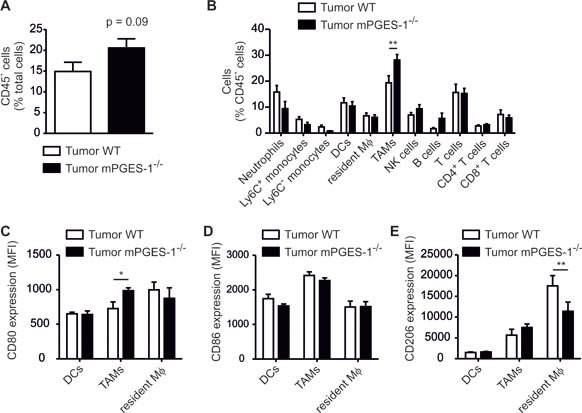
mPGES-1-deficiency increases the abundance of F4/80^+^ CD11c^+^ phagocytes in PyMT tumors PyMT mice were sacrificed 20 weeks after birth. Immune cell subsets and activation markers in PyMT tumors were determined by flow cytometry. (A) The relative content of CD45^+^ immune cells in tumors of wildtype (WT) and mPGES-1^−/−^ tumors is shown. (B) The relative amount of immune cell subsets is displayed. Data are means ± SEM of 11 WT and 7 mPGES-1^−/−^ mice. (C) CD80, (D) CD86, and (E) CD206 expression by tumor-associated macrophages (TAMs), dendritic cells (DCs), and resident macrophages (resident Mφ) in PyMT tumors is displayed. Data are means ± SEM of 6 WT and 7 mPGES-1^−/−^ mice. Exact *p*-values were calculated using student's t-test (A), otherwise *p-values* were calculated using two-way ANOVA with Bonferroni's correction (B-E). Asterisks indicate significant differences between experimental groups (*, *p* ≤ .05, **, *p* ≤ .01).

We asked whether reduced tumor outgrowth was correlated with a different immune status in the tumors. Hence, tumor single cell suspensions were analyzed by polychromatic FACS to characterize tumor-infiltrating leukocyte populations [25]. There was a tendency of increased leukocyte infiltration into mPGES-1-deficient tumors (Figure 6A). Phagocyte subpopulations in PyMT tumors are mainly F4/80^+^ CD11b^low^ CD11c^+^ tumor-associated macrophages (TAMs), CD11b^high^ F4/80^+^ resident macrophages, and F4/80^low^ CD11c^+^ MHCII^+^ DCs (Figure S1C) [28]. Furthermore, monocytes, neutrophils, and different lymphocyte populations are present (Figure 6B). In general, TAMs promote tumor growth and their abundance at the tumor site is often indicative of bad prognosis in breast cancer patients [29]. To our surprise, the relative content of TAMs was significantly increased in PyMT tumors of mPGES-1^−/−^ mice (Figure 6B). Thus, the usual correlation between breast tumor growth and the number of tumor infiltrating phagocytes was not reflected in our system. We hypothesized that, in agreement with our *in vitro* observations, polarization of TAMs in mPGES-1^−/−^ tumors might explain the differences in tumor growth. Therefore, polarization of phagocytes in WT and mPGES-1-deficient tumors was assessed using flow cytometry. When analyzing the immunophenotype of TAMs, we indeed observed an increase in CD80 expression in mPGES-1^−/−^ TAMs (Figure 6C). CD86 and CD206 expression in TAMs remained unchanged (Figure 6D,E). However, we observed reduced CD206 expression by resident macrophages in mPGES-1^−/−^ tumors. These data indicate a different impact of PGE_2_ on resident and monocyte-derived macrophages [28].

### MPGES-1-deficiency enhances phagocyte-dependent CTL activation

**Figure 7:**
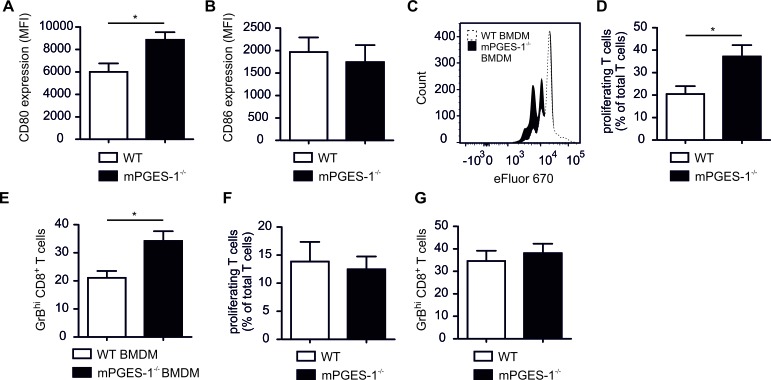
MPGES-1-deficiency promotes CTL activation by tumor-associated macrophages (A-E) Bone marrow monocytes of WT or mPGES-1^−/−^ mice were pre-stimulated with LPS/IFN-γ for 30 min and cocultured with E0771 tumor spheroids for 24 h. Afterwards, OVA SIINFEKL peptide was added for 1 h followed by addition of spleen-derived OT-I CTLs for another 4 days. Afterwards cocultures were analyzed by flow cytometry. (A) CD80 and (B) CD86 expression of CD11b^+^ F4/80^+^ monocyte-derived macrophages (BMDM) is displayed. Data are means ± SEM of four independent experiments. (C) A representative histogram and (D) a quantification of proliferating OT-I T cells is displayed. (E) The relative amount of GrB^hi^ CTLs is shown. Data are means ± SEM of four independent experiments using cells of 4 WT and 4 mPGES-1^−/−^ mice. (F,G) Pre-activated spleen-derived CTLs of WT or mPGES-1^−/−^ mice were cocultured with E0771 tumor spheroids for 3 days. (F) The relative amount of proliferating T cells and (G) the relative amount of GrB^hi^ CTLs is shown. Data are means ± SEM of four independent experiments using cells of 4 WT and 4 mPGES-1^−/−^ mice. *p*-values were calculated using student's t-test. Asterisks indicate significant differences between experimental groups (*, *p* ≤ .05).

Following the hypothesis that a reduced occurrence of mPGES-1^−/−^ PyMT tumors was due to enhanced immune control, we wondered if elevated CD80 expression in mPGES-1^−/−^ murine macrophages translates into CTL activation. First, we asked whether enhanced CD80 was observed in mPGES-1^−/−^ BMDM stimulated with LPS/IFN-γ compared to WT macrophages. Interestingly, mPGES-1^−/−^ BMDM, which barely produced PGE_2_, did not show alterations in CD80 or CD86 induction compared to WT BMDM after stimulation with LPS/IFN-γ (Figure S3C,D). Thus, not only an activating stimulus *per se*, but also the tumor milieu might be necessary to reveal immune modulating effects of PGE_2_. These selective immune modulating effects of PGE_2_ might be most apparent in a set-up that comprises both inflammatory (LPS/IFN-γ) as well as anti-inflammatory (breast tumor microenvironment) components. To test this hypothesis we generated tumor spheroids of E0771 murine mammary carcinoma cells. These spheroids were incubated with LPS/IFN-γ-stimulated WT or mPGES-1^−/−^ bone marrow monocytes for 24 h, followed by addition of OVA SIINFEKL as a model antigen and spleen-derived OT-I CTLs for another 4 days. These T cells specifically recognize the OVA SIINFEKL peptide, resulting in a model of antigen-specific CTL activation in the tumor microenvironment. In this setting, mPGES-1^−/−^ monocyte-derived tumor spheroid-infiltrating macrophages indeed expressed higher CD80, but not CD86, levels compared to wildtype macrophages (Figure 7A,B). These data corroborated our findings in the PyMT model. Spheroid-infiltrating OT-I cells displayed higher rates of proliferation (Figure 7C,D) and GrB expression (Figure 7E) when spheroids contained mPGES-1^−/−^ monocyte-derived macrophages compared to WT macrophages. These data support the notion that reduced tumor growth in mPGES-1^−/−^ PyMT mice was due to enhanced immune control. Importantly, infiltration of pre-activated WT or mPGES-1^−/−^ spleen CTLs did not affect T cell proliferation or GrB expression (Figure 7F,G). These novel data demonstrate that mPGES-1-deficiency does not affect basic CTL function. Thus, a direct impact of mPGES-1-deficiency on CTL function in PyMT tumors was unlikely contributing to the phenotype of mPGES-1^−/−^ PyMT mice. We conclude that mPGES-1^−/−^ TAMs support CTL activation. This might be an important mechanism in restricting tumor occurrence in mPGES-1^−/−^ PyMT mice.

## DISCUSSION

In our *in vitro* coculture tumor model, non-activated PBMCs were unable to kill tumor spheroids. Rather, they required TLR4 activation to increase CD80 expression and to concomitantly elevate GrB^hi^ CTL numbers. CD80 appeared not only as a signature marker, but also a functional prerequisite for immune activation in this model. Along these lines, blocking CD80 reduced the number of GrB^hi^ CTLs and prevented tumor spheroid killing. CD86 expression was not regulated by LPS and blocking of CD86 neither changed GrB^hi^ CTL numbers nor rescued tumor spheroid sizes. However, in the human system other phagocyte subsets might regulate CD86 expression in response to LPS. For instance, monocyte differentiation with human plasma generates CD14^+^ CD11c^+^ human macrophages that upregulate CD80 but not CD86, and a minor population of CD14^−^ CD11c^+^ cells that selectively upregulate CD86 upon LPS stimulation (data not shown).

Our *in vitro* model allowed investigating the immune modulating function of PGE_2_ in an authentic human tumor context. These functions were re-capitulated in the murine system both *in vitro* and *in vivo*. Due to the high reproducibility, this model might be easily adaptable for high-throughput analysis of compounds affecting tumor immunity. In this study, inhibition of mPGES-1 by C3 selectively inhibited PGE_2_ production. Importantly, it did not provoke prostanoid shunting as previously observed in LPS/C3-stimulated mouse peritoneal macrophages [11]. Shunting was also not observed in mPGES-1^−/−^ PyMT tumors. Either selective inhibition of PGE_2_ with C3 or inhibition of total prostanoid production with Cxb enhanced CD80 expressed on spheroid-infiltrating phagocytes. Thus, PGE_2_ was the relevant prostanoid responsible for modulating CD80 expression. Moreover, at least in the *in vitro* system, mPGES-1 inhibition recapitulated the effect of COX2 inhibition. Addition of exogenous PGE_2_, the EP2 receptor agonist butaprost, or the PDE4 inhibitor rolipram revealed that the PGE_2_/EP2/cAMP pathway impaired both CD80 and CD206 expression on phagocytes. We conclude that PGE_2_ signaling alters polarization of human tumor-associated phagocytes independent of the M1/M2 paradigm [30]. In line, PGE_2_ inhibited inflammatory macrophage function in numerous inflammation models through EP2/EP4 and elevated intracellular cAMP [31-33].

Modulation of costimulatory molecule expression by PGE_2_ requires the tumor scenario as CD80 expression of mPGES-1^−/−^ BMDMs was similar to WT BMDMs in monocultures. Only when infiltrating tumor spheroids mPGES-1^−/−^ macrophages expressed elevated CD80 levels. Similarly, mPGES-1^−/−^ GM-CSF/IL-4-differentiated bone marrow-derived dendritic cells (BMDCs) did not show altered levels of CD80 and CD86 after LPS/IFN-γ challenge, whereas EP2/cAMP signaling rather enhanced CD80 and CD86 expression in TNF-α-differentiated BMDCs [16, 34]. In BMDMs, LPS-triggered CD40 expression was inhibited by the addition of PGE_2_, CD80 expression remained unaffected, while CD86 was induced [35]. However, PGE_2_ inhibited CpG-induced CD80 expression and IFN-α secretion in human plasmacytoid dendritic cells via EP2 and EP4 signaling [36]. These findings suggest that regulation of costimulatory molecules in monocultures of BM-derived cells depends on the cell type, stimulus, and differentiation protocols. Principally, mPGES-1-deficiency does not seem to alter functional outcomes in phagocyte monocultures. We propose that monoculture models are suitable to investigate the induction of signaling molecules such as CD80/CD86. However, their fine-tuning often relies on more complex interactions e.g. a crosstalk of different cell types and/or the presence of tumor environmental factors. These factors are only available in tumor spheroid immune cell cocultures or *in vivo* tumor models. For instance, regulatory T cells may be involved in the regulation of CD80 and CD206 expression of phagocytes. PGE_2_ promotes Treg activity, which can directly regulate CD80/CD86 expression [37, 38].

In MMTV/PyMT mice, we observed that the loss of mPGES-1 delayed breast cancer development. In a different breast cancer model, Her2/c-neu mice, genetic depletion of mPGES-1 reduced mammary tumorigenesis, aromatase activity, and angiogenesis [39]. However, the impact on tumor-associated immunity was not investigated. We hypothesize that delayed breast tumor development in our model was due to prolonging tumor dormancy, indicating a potential of mPGES-1 inhibitors for tumor prophylaxis. An explanation for enhanced tumor dormancy might be a superior tumor immune control by CTLs, similar to the situation observed in our *in vitr*o murine model (that mimics early tumors). Nevertheless, we observed higher expression of CD80, a feature of immune control, also in advanced tumors. This feature may be more pronounced or relevant in small, dormant malignancies. Accordingly, although CD80-expressing mPGES-1^−/−^ macrophages more efficiently activated CTLs *in vitro*, the T cell infiltrate in established mPGES-1^−/−^ PyMT tumors was not altered. Thus, our data indicate that a deficient mPGES-1-dependent PGE_2_ production alone was insufficient to lift immune suppression from established PyMT tumors. *In vitro*, high expression of CD80 on human and murine phagocytes required TLR stimulation. Indeed, the FDA recently approved the TLR2/TLR4 agonist *Bacillus Calmette-Guérin* (BCG), the TLR2/TLR4 agonist monophosphoryl lipid A (MPL) and the TLR9 agonist imiquimod as anti-tumor agents [3]. The tumor vaccine L-BLP25, which contains an MPL component, failed to increase overall survival in a Phase III clinical trial in non-small cell lung carcinoma (NSCLC) patients. However, it showed a significant improvement in patients treated with low-dose cyclophosphamide, which is known to abrogate tolerance of regulatory T cells in tumor patients [40, 41]. Together with our findings we hypothesize that TLR agonists may not lack efficiency in general but their original anti-tumoral mode of action may be restricted by negative feedback mechanisms dependent, among others, on PGE_2_. Thus, the development of mPGES-1 inhibitors as natural combinational therapy partners for TLR agonists may be relevant to enhance the efficiency of TLR agonists as cancer therapeutics.

## MATERIALS AND METHODS

### Materials

The mPGES-1 inhibitor compound III (C3) (5 μM) [11] was synthesized by NovaSAID AB (Solna, Sweden). Celecoxib (5 μM), lipopolysaccharide (LPS) (50 ng/ml), dimethylsulfoxide (DMSO), and rolipram (10 μM) were purchased from Sigma-Aldrich (St.Louis, MO, USA). CD80 (5 μg/ml) and CD86 (5 μg/ml) neutralizing antibodies and the isotype control (5 μg/ml) were purchased from eBiosciences (Frankfurt, Germany) or R&D Systems (Minneapolis, MN, USA). PGE_2_, butaprost (5 μM), sulprostone (5 μM) and cay10580 (200 nM) were from Cayman Chemicals (Ann Arbor, MI, USA). Both human and murine interferon-γ (IFN-γ) (100 U/ml) were from Peprotech (Hamburg, Germany). Anti-CD3/anti-CD28 Dynabeads (1:20 - bead: PBMCs) were from Invitrogen (Carlsbad, Germany). OVA (257-267) SIINFEKEL was from Anaspec (Seraing, Belgium). Cell proliferation dye eFluor670 was from eBiosciences. Matrigel was from Corning Life Sciences (Amsterdam, Netherlands).

### Mouse strains and genotyping

All procedures involving mice followed the guidelines of the Hessian animal care and use committee. Wildtype (WT) and mPGES-1^−/−^ mice with or without crossing into a PyMT background as well as Rag1 knockout/transgenic OT-I T cell receptor (OT-I) mice (all C57BL/6) were used. For genotyping, tail-tips were lysed with KAPA Genotyping lysis buffer (Peqlab, Erlangen, Germany) and resulting DNA solutions were analyzed with PCR amplification using KAPA Hotstart Genotyping reaction mix (Peqlab) and standard protocols. Primers a and b were used for the wildtype allele and primers b and c were used for the mutated allele. ‘a’: 5′-CAG TAT TAC AGG AGT GAC CCA GAT GTG-3′ (specific for targeted mPGES-1 gene). ‘b’: 5′-GGA AAA CCT CCC GGA CTT GGT TTT CAG-3′ (specific for the mPGES-1 gene downstream of the targeting construct). ‘c’: 5′-ATC GCC TTC TAT CGC CTT CTT GAC GAG-3′ (specific for the neo resistance gene). Primers used for PyMT genotyping include PyMT forward: 5′-CTA GGC CAC AGA ATT GAA AGA TCT-3′ and PyMT reverse: 5′-GTA GGT GGA AAT TCT AGC ATC ATC C-3′. Internal control forward: 5′-GGA AGC AAG TAC TTC ACA AGG G-3′ and internal control reverse: 5′-GGA AAG TCA CTA GGA GCA GGG-3′.

### Cell culture and MCTS generation

MCF-7 breast carcinoma cells were purchased from ATCC-LGC Standard GmbH (Wesel, Germany). E0771 cells were from CH3 Biosystems (Amherst, NY, USA). Cells were cultured in RPMI 1640, supplemented with 5 mM glutamine, 100 U/ml penicillin, 100 μg/ml streptomycin as well as 10% heat-inactivated FCS and were maintained at 37°C in a humidified atmosphere with 5% CO_2_. Spheroids were generated from MCF-7 cells using the liquid overlay technique as described [21]. Spheroids generated from E0771 cells were initiated using 5 × 10^4^ cells/ml and were cultured in cell-repellent 96-well plates (Greiner Bio-One GmbH, Frickenhausen, Germany) with a final concentration of 1,5% matrigel [22].

### Isolation of human PBMCs

PBMCs were isolated from Buffy Coats of anonymous healthy donors obtained from DRK Blutspendedienst (Frankfurt, Germany) using Ficoll gradient centrifugation [23].

### Isolation of murine CD8^+^ T cells and monocytes

Spleens were isolated from WT, mPGES-1^−/−^, and OT-I mice. Single cell suspensions were generated by processing the spleen through a 70 μm nylon mesh (BD Biosciences, Heidelberg, Germany) with a syringe plug. After performing RBC lysis, CD8^+^ T cells were isolated using the mouse CD8^+^ T cell Isolation Kit (Stemcell Technologies, Cologne, Germany) and stained with the Cell Proliferation Dye eFluor670. Bone marrow was isolated from tibia and femur of WT and mPGES-1^−/−^ mice. After RBC lysis, monocytes were isolated using the Monocyte Isolation Kit and the AutoMACS separator (Miltenyi Biotec GmbH, Bergisch Gladbach, Germany).

### PBMC MCF-7 tumor spheroid cocultures

Media of MCF-7 spheroids were changed prior to PBMC addition. PBMCs were either left untreated or stimulated with 50 ng/ml LPS or LPS and 100 U/ml IFN-γ. Neutralizing antibodies or inhibitors were immediately added after activation of PBMCs. PBMCs were then cocultured with MCF-7 tumor spheroids for the times indicated. Tumor spheroid size was determined as described recently [21].

### E0771 tumor spheroid cocultures

For E0771 spheroid CD8^+^ T cell cocultures media were changed prior to the addition of 1 × 10^5^ CD8^+^ T cells per spheroid. CD8^+^ T cells were either left untreated or stimulated with anti-CD3/anti-CD28 Dynabeads (1:1 - bead:CD8^+^ T cell) for 30 min. E0771 spheroid mPGES-1^−/−^ and wildtype CD8^+^ T cell cocultures were maintained for 3 days. For cocultures of E0771 spheroids with monocytes and CD8^+^ OT-I T cells, 1 × 10^5^ monocytes prestimulated with 50 ng/ml LPS and 100 U/ml IFN-γ were added to each spheroid. 24 h after monocyte addition, cocultures were stimulated with OVA SIINFEKEL for 1 h, followed by addition of 1 × 10^5^ OT-I CD8^+^ T cells per spheroid. Cocultures were further maintained for 4 days.

### Prostanoid quantification by liquid chromatography-tandem mass spectrometry (LC-MS/MS)

LC-MS/MS analysis of PGF_2_α, PGE_2_, PGD_2_, and TXB_2_ from coculture supernatants and PyMT tumors was performed after solid-phase extraction and analysis was performed as previously described [24].

### Flow cytometry

Samples were acquired with a LSRII/Fortessa flow cytometer (BD Biosciences, Heidelberg, Germany) and analyzed using FlowJo software 7.6.1 (Treestar, Ashland, OR, USA) or FACSDiva (BD). All antibodies and secondary reagents were titrated to determine optimal concentrations. CompBeads (BD) were used for single-color compensation to create multi-color compensation matrices. For gating, fluorescence minus one (FMO) controls were used. The instrument calibration was controlled daily using Cytometer Setup and Tracking beads (BD). For staining of human spheroid-infiltrating phagocytes, cell suspensions were blocked with Fc Receptor Binding Inhibitor (eBioscience) for 15 min on ice and stained with anti-CD45-PE, anti-CD11c-V450, anti-CD14-APC-H7, anti-CD86-PE, anti-CD206-PE-Cy5 (BD), and anti-CD80-APC (eBioscience) for 15 min on ice. For murine spheroid-infiltrating phagocytes, anti-CD11b-eFluor605NC (ebioscience), anti-F4/80-PE-Cy7 (Biolegend), anti-CD80-APC and anti-CD86-PE (both BD) were used. For intracellular staining of GrB in human CTLs, non-infiltrating PBMCs were fixed with Cytofix/Cytoperm buffer (BD) for a maximum of 5 min on ice, followed by washing and permeabilization with Perm/Wash buffer (BD) as described [21]. Permeabilized cells were blocked with Fc Receptor Binding Inhibitor for 15 min on ice and GrB expression in CTLs was determined using anti-CD45-APC, anti-CD4-FITC, anti-GrB-PE (Immunotools, Friesoythe, Germany), anti-CD3-V450, anti-CD8-APC-Cy7 (BD). For mouse spheroid-associated CTLs, anti-CD3-PE-CF594 and anti-CD8-BV650 (both from BD) as well as anti-GrB-PE (ebioscience) were used. Characterization of PyMT tumor infiltrating leukocytes was performed essentially as described previously [25]. The following antibodies were used: anti-CD3-PE-CF594, anti-CD4-V500, anti-CD11c-AlexaFluor700, anti-CD19-APC-H7, anti-Ly6C-PerCP-Cy5.5 (all from BD), anti-CD8-eFluor650, anti-CD11b-eFluor605NC (both from eBioscience), anti-CD45-VioBlue, anti-CD49b-PE, anti-MHC-II-APC (all three from Miltenyi), anti-F4/80-PE-Cy7, anti-Ly6G-APC-Cy7, anti-SiglecH-FITC (all three from Biolegend, San Diego, CA, USA). To characterize phagocyte subset activation, PyMT tumor single cell suspensions were stained with anti-CD45-VioBlue (Miltenyi), anti-F4/80-PE-Cy7, anti-CD11c-PerCP-Cy5.5, anti-Ly6G-APC-Cy7 (Biolegend), anti-CD80-APC, anti-CD86-PE (BD), and anti-CD206-FITC (Biolegend).

### Screening of PyMT tumors

Female PyMT^+/−^ were screened from week 6 after birth for breast tumors. Tumor size (by caliper) and localization were monitored.

### Tissue isolation from PyMT mice and generation of single cell tumor suspensions

20 weeks after birth, PyMT mice were sacrificed and perfused with PBS. After perfusion, PyMT tumors and spleen were isolated and their respective weight was recorded. Tissues were lysed with the Miltenyi Tumor dissociation kit and the GentleMACS (Miltenyi, Bergisch Gladbach, Germany) using standard protocols.

### Statistical analysis

All data represented in graphs are means ± SEM unless stated otherwise. Statistically significant differences between groups were calculated using student's t-test or ANOVA with Bonferroni's post-correction and were considered significant if *, p ≤ 0.05, **, p ≤ 0.01, ***, p ≤ 0.001.

## SUPPLEMENTARY MATERIALS AND FIGURES


