# Supplementary material for: MPGES-1-derived PGE2 suppresses CD80 expression on tumor-associated phagocytes to inhibit anti-tumor immune responses in breast cancer
Source: Oncotarget. 2015 Mar 14;6(12):10284–96. doi: 10.18632/oncotarget.3581 (PMC4496355; doi:10.18632/oncotarget.3581)
Supplement: Supplementary file 1 [file oncotarget-06-10284-s001.pdf]

MPGES-1-derived PGE2 suppresses CD80 expression on tumor-associated phagocytes to inhibit anti-tumor immune responses in breast cancer

Supplementary Material

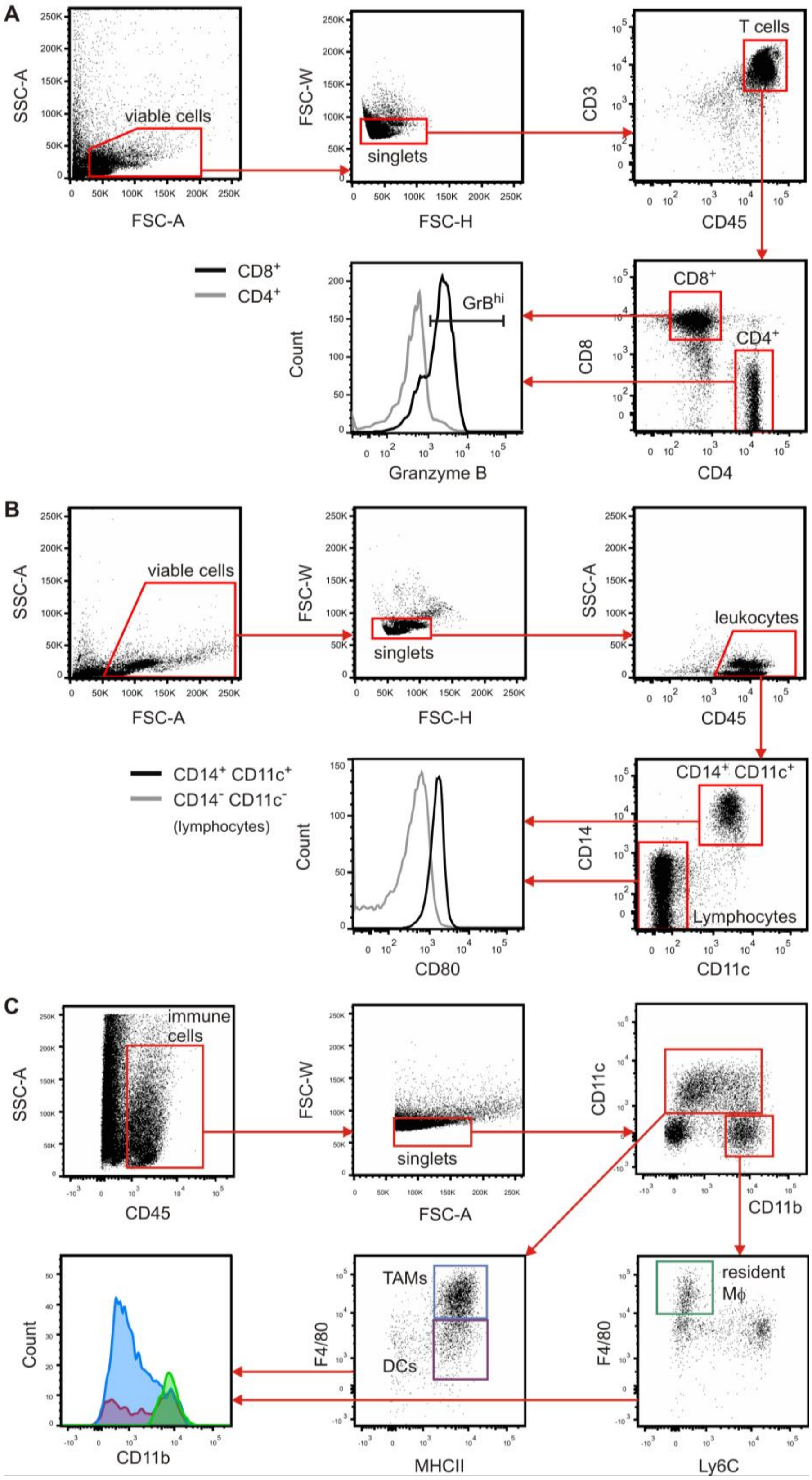

Figure S1: (A) Gating strategy for discrimination of T cell subsets and intracellular detection of their respective Granzyme B expression. PBMCs were isolated out of PBMC MCF-7 spheroid co-cultures and prepared for intracellular staining as described in Methods. First, viable single cells were identified. Single cells were classified as T cells ($CD45^+CD3^+$), T_H cells ($CD45^+CD3^+CD4^+$), and cytotoxic T cells ($CD45^+CD3^+CD8^+$). Granzyme B was stained in T cell subsets to determine the functionality of respective T cell subsets.

(B) Gating strategy for discrimination of $CD14^+$ $CD11c^+$ phagocytes in PBMC MCF-7 co-cultures and detection of their respective polarization markers. PBMCs were isolated from PBMC MCF-7 spheroid co-cultures as described in Methods. First, viable single cells were identified. Single cells were classified as leukocytes ($CD45^+$), $CD45^+CD14^+CD11c^+$ phagocytes, and $CD45^+CD14^-CD11c^-$ lymphocytes. Extracellular CD80, CD86, and CD206 were stained to characterize polarization of $CD14^+$ $CD11c^+$ phagocytes.

(C) Gating strategy for the discrimination of phagocyte subsets in PyMT breast tumors. PyMT mice were sacrificed 20 weeks after birth and tumor suspension cells were isolated from PyMT breast tumors as described in Methods. First, single immune cells were identified by gating on $SSC\text{-}A^{low}$ $CD45^+$ and $FSC\text{-}W^{low}$, $FSC\text{-}H^{int}$ cells. These were subgated as $CD11c^+CD11b^{low}F4/80^+$ tumor-associated macrophages (TAMs), $F4/80^-CD11c^+MHCII^+$ DCs and $F4/80^+CD11c^-CD11b^{high}$ resident macrophages (M□).

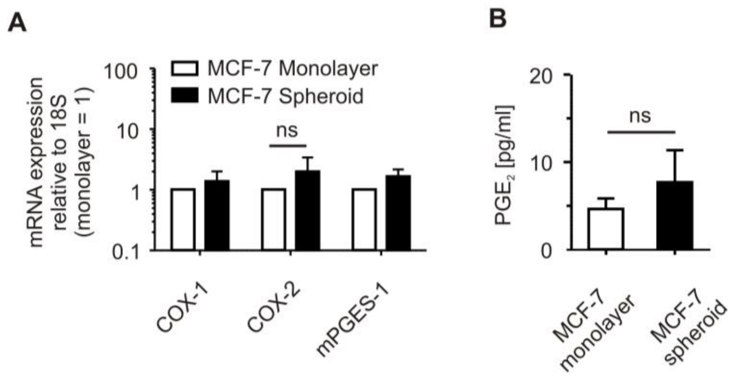

Figure S2: (A-B) MCF-7 cells were detached and seeded on agarose to generate tumor spheroids. (A) mRNA expression of PGE_2 metabolizing enzymes in monolayer cells or five days old tumor spheroids was analyzed using qPCR and (B) PGE_2 levels in MCF-7 culture supernatants were determined by PGE_2 EIA. Data are means ± SD of four independent experiments. Data were analyzed using GraphPad Prism 5.0. *p*-values were calculated using one-way ANOVA (A) or two-way ANOVA (B-C) with Bonferroni's correction. Asterisks indicate significant differences between experimental groups (*, $p < .05$, **, $p < .01$, ***, $p < .001$)

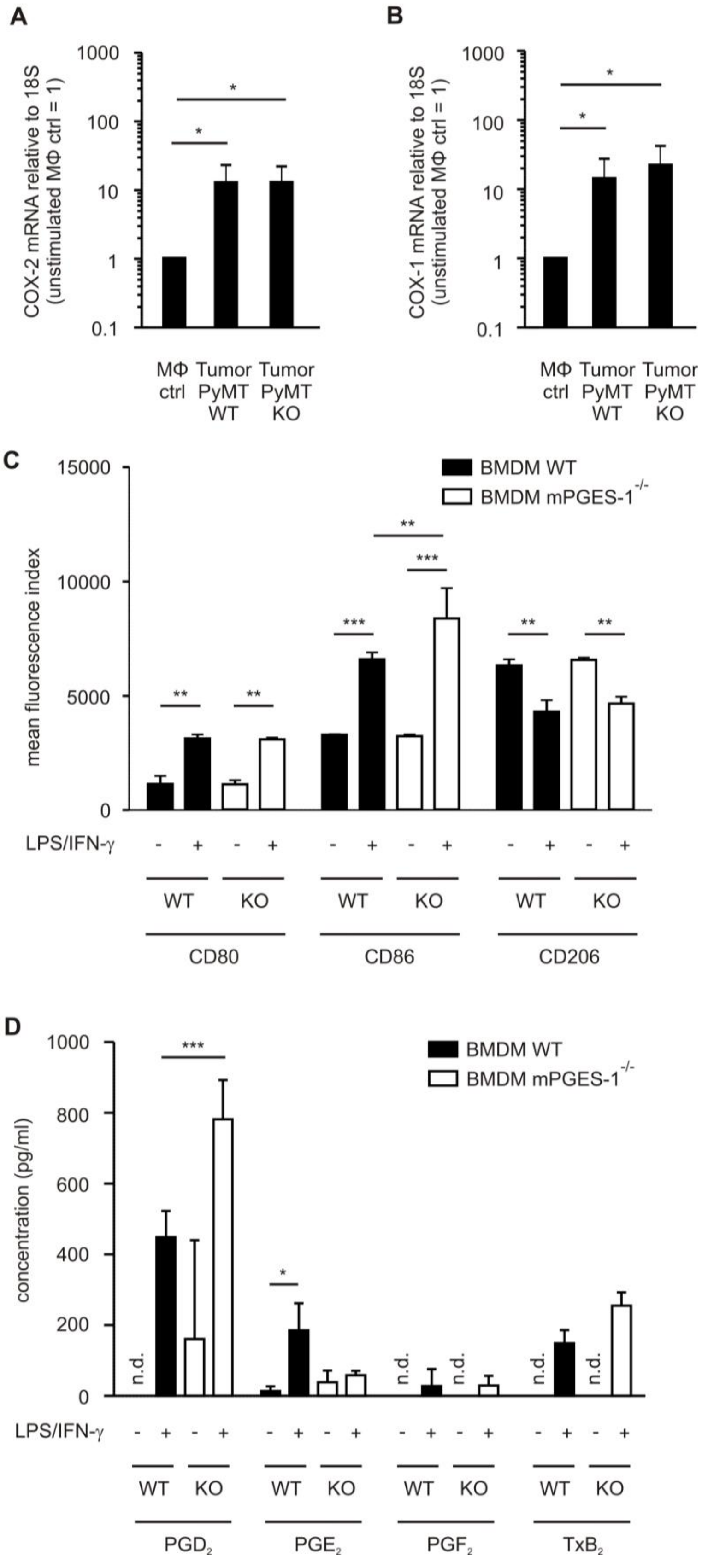

A
COX-2 mRNA relative to 18S (unstimulated MΦ ctrl = 1)
1000
100
10
1
0.1
*
*
MΦ ctrl
Tumor PyMT WT
Tumor PyMT KO
B
COX-1 mRNA relative to 18S (unstimulated MΦ ctrl = 1)
1000
100
10
1
0.1
*
*
MΦ ctrl
Tumor PyMT WT
Tumor PyMT KO
C
BMDM WT
BMDM mPGES-1-/-
mean fluorescence index
15000
10000
5000
0
**
**
***
**
***
**
**
LPS/IFN-γ
- + - + - + - + - + - +
WT KO WT KO WT KO
CD80
CD86
CD206
D
BMDM WT
BMDM mPGES-1-/-
concentration (pg/ml)
1000
800
600
400
200
0
***
*
n.d.
LPS/IFN-γ
- + - + - + - + - + - + - + - +
WT KO WT KO WT KO WT KO
PGD2
PGE2
PGF2
TxB2

Figure S3: (A-B) PyMT mice were sacrificed 20 weeks after birth. mRNA expression of (A) COX-2 and (B) COX-1 in PyMT tumors and unstimulated BMDM macrophages was analyzed using qPCR. (C-D) Bone marrow-derived macrophages were isolated using standard protocols and stimulated with LPS/IFN-□ for 8h. (C) Prostanoids PGE_2, PGD_2, $PGF_{2\square}$ and TxB_2 in supernatants of PBMC MCF-7 spheroid co-cultures were measured by LC-MS/MS and (D) Polarization markers of $CD14^+$ $CD11c^+$ phagocytes is displayed. Data are means ± SD of at least four independent donors. Data were analyzed using GraphPad Prism 5.0. p-values were calculated using one-way ANOVA (A,B) or two-way ANOVA (C,D) with Bonferroni's correction. Asterisks indicate significant differences between experimental groups (*, p < .05, **, *p* < .01, ***, *p* < .001)
